# Actual 10-Year Survival after Resection of Perihilar Cholangiocarcinoma: What Factors Preclude a Chance for Cure?

**DOI:** 10.3390/cancers13246260

**Published:** 2021-12-13

**Authors:** Anne-Marleen van Keulen, Pim B. Olthof, Matteo Cescon, Alfredo Guglielmi, William R. Jarnagin, Silvio Nadalin, Johann Pratschke, Francesca Ratti, Roberto I. Troisi, Bas Groot Koerkamp, Stefan Buettner, Joris I. Erdmann, on behalf of the ‘Perihilar Cholangiocarcinoma Collaboration Group’

**Affiliations:** 1 Erasmus Medical Center, Department of Surgery, 3015 GD Rotterdam, The Netherlands; annemarleenvankeulen@gmail.com (A.-M.v.K.); b.grootkoerkamp@erasmusmc.nl (B.G.K.); s.buttner@erasmusmc.nl (S.B.); 2 Department of Surgery, Amsterdam University Medical Centers, 1105 AZ Amsterdam, The Netherlands; j.i.erdmann@amsterdamumc.nl; 3 General Surgery and Transplantation Unit, Department of Medical and Surgical Sciences, University of Bologna, 40126 Bologna, Italy; matteo.cescon@unibo.it; 4 Unit of Hepato-Pancreato-Biliary Surgery, Department of Surgery, University of Verona Medical School, 37134 Verona, Italy; alfredo.guglielmi@univr.it; 5 Department of Surgery, Memorial Sloan-Kettering Cancer Center, New York, NY 1275, USA; jarnagiw@mskcc.org; 6 Department of General, Visceral and Transplant Surgery, University Hospital Tübingen, 72076 Tübingen, Germany; silvio.nadalin@med.uni-tuebingen.de; 7 Department of Surgery, Charité—Universitätsmedizin Berlin, 13353 Berlin, Germany; johann.pratschke@charite.de; 8 Hepatobiliary Surgery Division, San Raffaele Hospital, 20158 Milan, Italy; ratti.francesca@hsr.it; 9 Department of Clinical Medicine and Surgery, Federico II University Hospital Naples, 80131 Napoli, Italy; Roberto.troisi@ugent.be

**Keywords:** perihilar cholangiocarcinoma, Klatskin tumor, surgery, survival, prognosis, cure

## Abstract

**Simple Summary:**

Long-term survival for patients with perihilar cholangiocarcinoma (pCCA) is rare. The median overall survival of patients undergoing curative-intent surgery for pCCA is 19 to 39 months. This multicenter study aimed to determine the cure rate and to identify clinicopathological factors that may preclude cure. Four hundred and sixty patients were included with a median follow-up of 10 years. Median OS was 29.9 months. Twenty-nine (6%) patients reached 10-year OS. The observed cure rate was 5%. Factors that virtually precluded cure (i.e., below 1%) according to the mixture cure model included age above 70, Bismuth–Corlette type IV tumors, hepatic artery reconstruction, and positive resection margins. Cure was unlikely (i.e., below 3%) in patients with positive lymph nodes or poor tumor differentiation. These factors need to be considered in patient counseling and long-term follow-up after surgery.

**Abstract:**

Complete resection of perihilar cholangiocarcinoma (pCCA) is the only potentially curative treatment. Long-term survival data is rare and prognostic analyses are hindered by the rarity of the disease. This study aimed to determine the cure rate and to identify clinicopathological factors that may preclude cure. All consecutive resections for pathologically confirmed pCCA between 2000 and 2009 in 22 centers worldwide were included in a retrospective cohort study. Each center included its retrospective data series. A total of 460 patients were included with a median follow-up of 10 years for patients alive at last follow-up. Median overall survival (OS) was 29.9 months and 10-year OS was 12.8%. Twenty-nine (6%) patients reached 10-year OS. The observed cure rate was 5%. Factors that virtually precluded cure (i.e., below 1%) according to the mixture cure model included age above 70, Bismuth-Corlette type IV tumors, hepatic artery reconstruction, and positive resection margins. Cure was unlikely (i.e., below 3%) in patients with positive lymph nodes or poor tumor differentiation. These factors need to be considered in patient counseling and long-term follow-up after surgery.

## 1. Introduction

Perihilar cholangiocarcinoma (pCCA) is a rare malignancy that manifests at or near the biliary confluence and accounts for 50–60% of all cholangiocarcinomas [1]. Patients usually present with the sequelae of biliary obstruction, such as painless jaundice and intrahepatic biliary dilatation on diagnostic imaging [2]. Patients and clinicians are confronted with many challenges from initial presentation until the definitive treatment. Obstacles faced range from confirming malignancy to biliary drainage with the risk of subsequent cholangitis. The majority of patients are eventually not amenable to surgery because of locally advanced or metastatic disease at the time of presentation [3]. This leaves the possibility of resection reserved for less than half of all patients (33.0–41.3%) [2,3,4].

Realistically, cure is rare, and only possible after extrahepatic bile duct resection in combination with partial hepatectomy with negative resection margins and locoregional lymphadenectomy. Over the past decades, significant improvement in long-term survival after surgery has been observed for patients diagnosed with pCCA that can be attributed to advances in surgical management [5,6,7]. Patients undergoing curative-intent surgery for pCCA have a median overall survival of 19 to 39 months, with 20–47% 5-year survival rates [8]. Due to the aggressive biology of the disease, survival rates remain poor with early hematogenous, lymphatic, and perineural dissemination of cancer cells [9,10,11].

Although several perioperative outcome parameters after curative-intent surgery for pCCA have been associated with long-term survival, a definition of cure after resection of pCCA is lacking. For colorectal liver metastases, a more pragmatic definition of cure is set at 10-year disease-free survival (DFS) [12]. This study aimed to determine the cure rate defined as 10-year overall survival (OS) with no evidence of recurrence at last follow-up after resection of pCCA and to identify clinicopathological factors that preclude cure in a large international multicenter cohort.

## 2. Methods

A total of 22 centers worldwide included all consecutive resections for pathologically confirmed pCCA after the year 2000. The period of inclusion varied across centers. For the aims of this study, patients who underwent surgery between 2000 and 2009 were selected, to ensure that a total follow-up of at least 10 years could potentially be observed. pCCA was defined as a biliary tumor originating at the hepatic duct confluence between the segmental bile ducts and cystic duct. Using a standardized and anonymized data file, each center comprised its retrospective data series. Patients were excluded in case they had only undergone explorative laparotomy or liver transplantation. Ethical approval was waived by the Institutional Medical Ethics Committee of the Amsterdam University Medical Center.

### 2.1. Patient Work-Up and Management

Work-up and perioperative management, such as patient selection for portal vein embolization (PVE) and biliary drainage, differed across centers due to the multicenter set-up. Patient selection for PVE and biliary drainage, therefore, differed between centers. Basically, most patients planned for large liver resections underwent preoperative, endoscopic, or transhepatic biliary drainage of at least the future liver remnant (FLR). Approaches to portal vein reconstruction vary across centers, as some perform it routinely whereas others perform it on demand.

### 2.2. Definitions

The definition of cure was 10-year overall survival with no evidence of recurrence at last follow up. OS was defined as the time elapsed between surgical resection and death of disease or last follow-up. OS is a more reliable and more objectively determinable endpoint than disease-specific survival (DSS).

Preoperative cholangitis was characterized as fever and leukocytosis requiring biliary drainage [13,14]. Resection of at three or more Couinaud liver segments was considered a major liver resection. Pathology records describing R0 resection margins were defined as tumor-free margins, indicating no evidence of tumor cells at any of the reported resection margins of the resected specimen. The absence of cancer cells in regional lymph nodes was indicated as N0 (or ‘negative’) nodal status. N1 (or ‘positive’) nodal status indicates that the cancer has spread to 1 or more lymph nodes.

### 2.3. Statistical Analysis

Survival rates were calculated using the Kaplan–Meier method. Differences in patient and disease characteristics between OS groups (<2 years, 2–5 years, 5–10 years, >10 years) were presented. Patients who were lost to follow-up prior to 10 years were excluded from the respective survival cohorts. After extended follow-up, the proportional hazards assumption of Cox proportional hazard analyses fails. We, therefore, employed a semi-parametric mixture cure model, in order to predict long-term survivors [12]. Truly cured patients from the cohort were selected and compared to the predicted cured patients using the semi-parametric mixture cure model. Categorical variables were described as counts and percentages. *p* < 0.05 was considered significant.

## 3. Results

### 3.1. Patient Characteristics

A median of 80 (range: 25–115) resections for pCCA per center were included. The baseline and (pre)operative characteristics across different survival cohorts are presented in Table 1. A total of 1667 patients made up the total cohort. From these, 460 patients resected between 2000 and 2009 were enrolled in the study population and underwent combined liver and biliary resection for histopathologically confirmed pCCA.

The majority of patients presented with jaundice (*n* = 318, 80%), with the consequent need for preoperative drainage in most patients (*n* = 381, 83%). A total of 77 (18%) patients suffered from preoperative cholangitis. Eighty patients (17%) underwent PVE prior to surgical resection. Intraoperative vascular reconstruction was performed more frequently for the portal vein (*n* = 138, 30%) when compared to hepatic artery reconstruction (*n* = 10, 2%). Pancreatoduodenectomy was performed in 5 patients (1%). Tumor-free margins were microscopically confirmed in 304 (67%) patients. In 214 (48%) patients, the tumor was classified as T3 or T4 according to the AJCC staging system (7th edition) [15]. Positive lymph nodes were found in 170 (38%) patients. Perineural invasion was present in 241 patients (72%). Tumor differentiation was poor in 99 patients (23%).

### 3.2. Overall Survival

**Figure 1:**
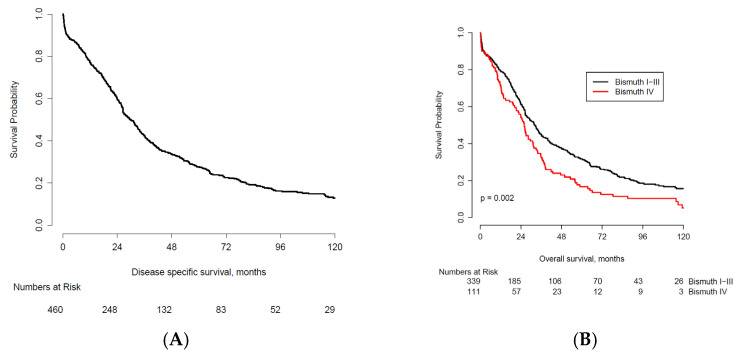

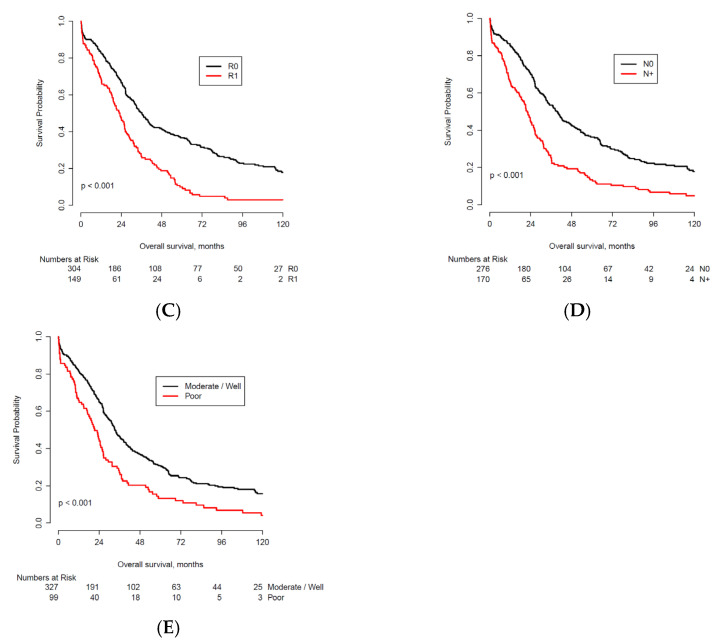
(**A**). Kaplan–Meier plot of OS for 460 patients undergoing resection for pCCA between 2000 and 2009. (**B**). Kaplan-Meier OS curves comparing survival by Bismuth-Corlette tumor classification. (**C**). Kaplan–Meier OS curves comparing survival by R0 and R1 resection margins. R0 resection indicates a microscopically margin-negative resection. R1 resection indicates the removal of all macroscopic disease, but microscopic margins are positive for tumor. (**D**). Kaplan–Meier OS curves comparing survival by lymph node status. (**E**). Kaplan–Meier OS curves comparing survival by tumor differentiation.

Figure 1A demonstrates the overall survival for the entire cohort (*n* = 460). The median follow-up was 10 years (119 months). At last follow-up, 362 patients (79%) had died. Median OS was 29.9 months and 10-year OS was 12.8% (95% CI: 9.6–16.6). Differences in long-term survival were observed for several prognostic factors: Bismuth-Corlette classification type (HR 1.45, 95% CI 1.15–1.83; Figure 1B), resection margins (HR 2.01, 95% CI 1.61–2.51; Figure 1C), lymph node metastasis (HR 1.98, 95% CI 1.59–2.46; Figure 1D), and tumor differentiation (HR 1.71, 95% CI 1.34–2.18; Figure 1E).

Ten-year OS was higher when comparing: Bismuth-Corlette I-III type tumor (*n* = 26, 7.7%) versus type IV tumors (*n* = 3, 2.7%), R0 resection (*n* = 27, 8.9%) versus R1 resection (*n* = 2, 1.3%), negative lymph node status (*n* = 24, 8.7%) versus positive lymph node status (*n* = 4, 2.4%), and poor tumor differentiation (*n* = 3, 3.0%) versus moderately/well tumor differentiation (*n* = 25, 7.6%).

### 3.3. Actual 10-Year Survivors

A total of 29 patients (6%) reached 10-year OS. The most important prognostic factors of these patients are summarized in Supplementary Table S1. Among the 10-year survivors, the majority was still alive without evidence of disease (*n* = 20, 69%), five patients (17%) died without evidence of disease after 10 years. Four patients (14%) developed recurrence within 10 years and were alive with disease 10 years after their initial resection. None of these patients underwent pancreatoduodenectomy or a portal vein or hepatic artery reconstruction. Upon final pathology, all four patients had negative resections margins, one patient had positive lymph nodes, three patients had perineural invasion, and tumor differentiation was moderate for all four.

### 3.4. Observed Cure and Cure Model

Twenty-five patients (5%) reached 10-year recurrence-free survival and were therefore considered cured (Table 2). The lowest observed cure rates for preoperative factors included age > 70 (4/131, 3.1%), and Bismuth-Corlette IV tumors (3/111, 2.7%). The lowest observed cure rates for operative factors included portal vein reconstruction (7/138, 5.1%), and hepatic artery reconstruction (0/10, 0%). The lowest observed cure rates for factors known postoperatively included positive resection margins (2/149, 1.3%), positive lymph nodes (3/170, 1.8%), and poor tumor differentiation (3/99, 3%). The observed rates were comparable with the calculated values from the mixture cure model with the following rates for preoperative factors: 0% when age >70, and 0% for Bismuth-Corlette IV tumors. The following rates for operative factors: 4.6% for portal vein reconstruction, and 0% for hepatic artery reconstruction. For postoperatively known factors the rates were: 0.9% for positive resection margins, 2.5% for positive lymph nodes, and 3% for poor tumor differentiation. No patient survived beyond 10 years postoperatively in case a hepatic artery reconstruction was performed.

## 4. Discussion

In a large international cohort, we found an observed cure rate (i.e., 10-year OS without evidence of recurrence at last follow-up) of 5% after resection of pCCA. According to the mixture cure analysis, several factors virtually precluded cure (i.e., below 1%) including age above 70, Bismuth-Corlette IV tumors, hepatic artery reconstruction, and positive resection margins. Cure was very unlikely (i.e., below 3%) in patients with positive lymph nodes or poor tumor differentiation.

Surgery is considered the only potentially curative treatment for pCCA. Previous studies that aimed at predicting survival after resection have repeatedly identified prognostic factors such as resection margin, tumor differentiation, lymph node involvement, vascular infiltration, and presence of metastasis [16,17,18]. Achieving negative resection margins is considered the main goal of surgery. The importance of reaching R0 over R1 margins is illustrated by a higher 5-year survival rate of 47% in case negative margins were achieved, compared to 21.9% for R1/R2 resections [17]. The current study confirmed previous observations where R0 resections were found crucial for potential cure, as the observed and predicted cure rates were 7.1% and 14.6%, respectively, for negative resection margins, achieving the highest survival rates in this study. This knowledge justifies the aggressive surgical approach in undertaking extended resections, considering that pCCA has a strong propensity to progress along the biliary tree and penetrate the surrounding tissues and vasculature. However, a positive margin may also simply reflect aggressive disease that is more advanced than anticipated on imaging preoperatively, and actually incurable by surgery.

Along with resection margins, lymph node involvement is considered a critical factor for prognostic evaluation [6,19]. This may occur at an early stage and is associated with poor survival with survival rates of 22.3% at 5 years for pCCA [20]. This corresponds with the current study, with observed and predicted 10-year disease-free survival of 1.8% and 2.5%, respectively, in the case of positive lymph nodes. Although it is currently well established that regional lymphadenectomy is required for staging, optimal strategies concerning the extent of lymph node dissection and its therapeutic value remain a topic of debate. Earlier studies focused primarily on the extent of lymphadenectomy (extended versus locoregional). However, more recently, attention has been drawn to the required numerical lymph node count to secure representative staging [21]. Insufficient numbers of retrieved lymph nodes may underestimate the extent of disease. Consequently, when insufficient lymph nodes have been retrieved patients are incorrectly classified as having N0 disease. The American Joint Committee on Cancer (AJCC) staging manual stratifies lymph node involvement into three groups, of which subgroup N2 handles a cutoff point of four positive lymph nodes. However, no statement is included regarding the minimally required number of retrieved lymph nodes. As for now, lymph node count ≥ 7 and lymph node ratio (ratio between positive lymph nodes and the total number of retrieved lymph nodes) are proposed as the most optimal benchmarks to identify lymph node metastases as a major prognostic factor for survival after resected pCCA [21,22].

In the current study, six remaining factors were also related to survival rates below 5%, specifically age ≥ 70, Bismuth-Corlette IV tumors, hepatic artery or portal vein reconstruction, tumor differentiation, and pancreatoduodenectomy. As seen in the OS subgroups, the majority of patients with these characteristics died within the first two years of follow-up. Most of the previously mentioned factors were associated with large resections and came with high operative risk. Moreover, in clinical practice, the impact of major surgery continues to have its effects after discharge. Recovery after surgery, readmissions, and recurrence in the first postoperative years all take a toll on the patient. Therefore, it is of importance to preoperatively optimize patients’ conditions. Preoperative procedures such as portal vein embolization (PVE) or associating liver partition and portal vein ligation for staged hepatectomy (ALPPS) have shown their value in increasing future liver remnant and reducing liver failure and 90-day mortality [23,24]. These procedures, however, are susceptible to patient selection and not all patients reach surgery after PVE or the first stage of ALPPS. Age, however, is a non-modifiable factor. Advanced age by itself is not a contra-indication for surgery, and several studies showed that surgery for pCCA can be performed with low mortality rates [25,26]. However, comorbidities are more likely to be present in older patients, and the rate of liver regeneration is diminished [27], which is key following major hepatectomy. Careful patient selection and preoperative assessment are essential in general, but specifically in the older population, to achieve acceptable postoperative outcomes.

Although surgery comes with risks, the prognosis of palliative treatment of pCCA is dismal, with a median survival of less than 6 months [28]. Therefore, accepting high operative risk may be justified in these patients. Although major improvements have been made, the future challenge consists of further improving preoperative assessment and patient selection to identify which patients will benefit from extensive surgery. At several stages (preoperative, operative, and postoperative), the factors in this study can aid the patient and the surgeon with decision making. For instance, a patient aged > 70 with intraoperatively evident lymph node metastasis, who requires vascular resection to achieve questionable negative margins will likely end up with dismal long-term survival, which should be weighed against the operative risk of such a complex resection in that specific patient. However, it should be noted that when left unresected, survival may be even more dismal with all the sequelae of cholangitis and failing biliary drainage procedures [29].

The studied prognostic factors in this study have been reported in previous literature. Presumed insights that are confirmed by this study include that 10-year survival is not realistic in patients with resectable pCCA. Considering the low 10-year survival rates and the limited differences seen across the presence or absence of most risk factors for survival in this study, perhaps the most effective measure to increase survival is to limit postoperative mortality. First, by improving surgical and oncological risk assessment (functional testing of general condition and liver remnant function), and second, by employing preoperative strategies to improve outcomes (e.g., PVE, prehabilitation, neoadjuvant therapy). Prehabilitation beholds preoperative interventions to improve patients’ health and fitness to improve postoperative outcomes [30,31]. A recent systematic review on the effect of prehabilitation on postoperative complications after hepatopancreatobiliary surgery showed a trend towards complication reduction. Interpretation should be carried out with caution due to the large variety in the rehabilitation programs, the indications and extent of liver surgery, and studied outcomes measures [32]. A randomized study on prehabilitation before elective liver resection demonstrated that a 4-week prehabilitation program improved preoperative cardiopulmonary exercise tests prior to liver surgery. Although postoperative mortality was not included in the analysis, improved cardiopulmonary function is associated with lower mortality after major abdominal surgery [33].

This study should be viewed in light of several limitations. Even though our cohort is one of the largest of its kind, the sample size in patient subgroups and in the 10-year survivor group was limited, possibly reducing the accuracy of the survival estimates. For example, patients lost to follow-up prior to 10 years were excluded from the survival cohorts. Therefore, results will probably be relatively underestimated and true survival will be higher. However, loss to follow-up is around 20% and therefore quite limited. As in all retrospective analyses, selection bias and confounding by indication were certainly present. Follow-up was not standardized; recurrences may have been missed. With respect to some clinicopathological factors, there is a tendency for a higher predicted cure when compared to an observed cure. This might have resulted from an insufficient period of follow-up, as not all patients were followed up for 10 years. Another limitation is the duration of the study period, introducing possible changes in treatment and management. For example, the low use of PVE could be explained because by the more liberal approach to PVE in recent years. Last, there are limitations in the definition of cure, which was derived from a study on cure after resection of colorectal liver metastasis [12]. In the current definition, the cause of death is ignored by the use of ‘overall’ survival. A patient experiencing a fatality at 9 years postoperatively that is not related to pCCA is defined uncured according to the current definition, although most physicians would argue this to be an excellent result. Unfortunately, the cause of death was not available in this multicenter study and could, therefore, not be used for analysis. The current definition is justified because pCCA is the cause of death in the majority of patients (91%) [34].

## 5. Conclusions

Despite considerable improvements made in surgical technique and perioperative management, long-term survival after surgery for pCCA remains poor. The invasive biology of pCCA challenges the more or less exhausted surgical resources to improve locoregional control. Identification of unfavorable prognostic factors is unlikely to deny a patient a resection, however, will rather be used to improve patient counseling and determine the need for (neo) adjuvant therapy and close long-term surveillance.

## Figures and Tables

**Table 1:** Baseline and (pre) operative characteristics across different survival cohorts.

Characteristic		*n*	<2 Years *	%	2-Year OS Probability †	2–5 Years *	%	5-Year OS Probability †	5–10 Years *	%	10-Year OS Probability †	>10 Years	%
*n*		460	177	38.5		129	28.0		50	10.9		29	6.3
Preoperative Factors													
Sex	Female	192	70	36.5	61.7	56	29.2	27.9	19	9.9	14.1	14	7.3
	Male	268	107	39.9	58.4	73	27.2	27.4	31	11.6	11.9	15	5.6
Age ≥ 70 years	No	327	125	38.2	60.2	99	30.3	25.9	33	10.1	12.7	23	7.0
	Yes	131	52	39.7	58.4	30	22.9	31.8	17	13.0	13.2	6	4.6
Jaundice at presentation	No	81	21	25.9	72.5	21	25.9	43.1	15	18.5	17.3	5	6.2
	Yes	318	136	42.8	55.4	89	28.0	23.4	27	8.5	12	20	6.3
ASA classification ≥ 3	No	251	89	35.5	62.3	68	27.1	30.5	28	11.2	15.1	20	8.0
	Yes	170	75	44.1	54.5	43	25.3	25.3	19	11.2	10	7	4.1
Preoperative biliary drainage	No	79	26	32.9	64.7	19	24.1	38.5	17	21.5	7.6	3	3.8
	Yes	381	151	39.6	58.5	110	28.9	25.4	33	8.7	13.8	26	6.8
Preoperative cholangitis	No	344	133	38.7	59.4	92	26.7	27.9	38	11.0	12.4	20	5.8
	Yes	77	31	40.3	57.9	23	29.9	24.9	6	7.8	14.9	7	9.1
Bismuth- Corlette IV	No	339	124	36.6	61.4	86	25.4	31.7	40	11.8	15.7	26	7.7
	Yes	111	49	44.1	54.9	39	35.1	16.7	9	8.1	5.2	3	2.7
PVE performed	No	379	138	36.4	62.1	111	29.3	28.8	47	12.4	12.4	24	6.3
	Yes	80	39	48.8	48.4	18	22.5	21.8	3	3.8	15.3	5	6.3
Operative Factors													
Major resection	No	54	21	38.9	59.7	17	31.5	26	5	9.3	14.9	5	9.3
	Yes	406	156	38.4	59.6	112	27.6	27.9	45	11.1	12.5	24	5.9
PV reconstruction	No	322	115	35.7	62.5	89	27.6	31.3	41	12.7	13.7	22	6.8
	Yes	138	62	44.9	52.4	40	29.0	19.2	9	6.5	10.7	7	5.1
HA reconstruction	No	394	147	37.3	60.5	113	28.7	27.1	44	11.2	11.1	19	4.8
	Yes	10	6	60.0	40	3	30.0	10	1	10.0	0	0	0.0
Pancreatoduodenectomy	No	375	138	36.8	60.9	109	29.1	26.8	40	10.7	11.3	18	4.8
	Yes	5	4	80.0	20	1	20.0	0	0	0.0	0	0	0.0
Postoperative Factors													
Positive resection margins	No	304	98	32.2	66.5	80	26.3	36.7	42	13.8	17.9	27	8.9
	Yes	149	73	49.0	47.6	48	32.2	10.1	8	5.4	3	2	1.3
AJCC T3 or T4	No	229	66	28.8	70.5	69	30.1	37.1	32	14.0	17.6	20	8.7
	Yes	214	101	47.2	48.8	56	26.2	18.1	17	7.9	7.9	8	3.7
Positive lymph nodes	No	276	80	29.0	69.8	84	30.4	36.3	38	13.8	17.8	24	8.7
	Yes	170	87	51.2	44.8	44	25.9	13.4	11	6.5	4.8	4	2.4
Poor tumor differentiation	No	327	109	33.3	64.7	97	29.7	30.7	36	11.0	15.8	25	7.6
	Yes	99	52	52.5	45.1	28	28.3	13.2	7	7.1	4.1	3	3.0
Perineural invasion	No	96	22	22.9	75.9	22	22.9	42.6	11	11.5	21.5	9	9.4
	Yes	241	115	47.7	50.1	71	29.5	19.2	17	7.1	10.9	17	7.1

* Only deceased patients. † Kaplan–Meier estimate. ASA—American Society of Anesthesiologists, PVE—Portal vein embolization, PV—portal vein, HA—hepatic artery, AJCC—American Joint Committee on Cancer.

**Table 2:** Characteristics of patients with observed cure and probability of cure.

Characteristics		Full Cohort	Observed Cure *	%	Probability of Cure †
*n*		460	25	5.4	
Preoperative Factors					
Sex	Female	192	10	5.2	5.2
	Male	268	15	5.6	11.6
Age ≥ 70 years	No	327	21	6.4	10.7
	Yes	131	4	3.1	0.0
Jaundice at presentation	No	81	5	6.2	13.8
	Yes	318	17	5.3	8.1
ASA classification ≥ 3	No	251	19	7.6	10.6
	Yes	170	6	3.5	7.3
Preoperative biliary drainage	No	79	3	3.8	8.6
	Yes	381	22	5.8	9.1
Preoperative cholangitis	No	344	19	5.5	7.7
	Yes	77	6	7.8	13.7
Bismuth-Corlette IV	No	339	22	6.5	12.9
	Yes	111	3	2.7	0.0
PVE performed	No	379	21	5.5	9.0
	Yes	80	4	5.0	10.9
Operative Factors					
Major resection	No	54	5	9.3	8.2
	Yes	406	20	4.9	8.8
PV reconstruction	No	322	18	5.6	11.9
	Yes	138	7	5.1	4.6
HA reconstruction	No	394	15	3.8	9.3
	Yes	10	0	0.0	0.0
Pancreatoduodenectomy	No	375	14	3.7	-
	Yes	5	0	0.0	-
Postoperative Factors					
Positive resection margin	No	304	23	7.6	14.6
	Yes	149	2	1.3	0.9
AJCC T3 or T4	No	229	18	7.9	11.6
	Yes	214	6	2.8	7.0
Positive lymph nodes	No	276	21	7.6	13.9
	Yes	170	3	1.8	2.5
Poor tumor differentiation	No	327	21	6.4	11.7
	Yes	99	3	3.0	3.0
Perineural invasion	No	96	8	8.3	12.7
	Yes	241	14	5.8	8.1

* Observed cure is defined as 10-year survival without recurrence. † Probability of cure was estimated from semiparametric mixture cure models. ASA—American Society of Anesthesiologists, PVE—Portal vein embolization, PV—portal vein, HA—hepatic artery, AJCC—American Joint Committee on Cancer.

## Data Availability

Data was provided by the Perihilar Cholangiocarcinoma Collaboration Group. The data presented in this study are available on request from the corresponding author.

## Supplementary Materials

The following are available online at https://www.mdpi.com/article/10.3390/cancers13246260/s1, Table S1: provides an overview of the clinicopathological factors of the 29 patients that reached 10-year overall survival.
