# Supplementary material for: Actual 10-Year Survival after Resection of Perihilar Cholangiocarcinoma: What Factors Preclude a Chance for Cure?
Source: Cancers (Basel). 2021 Dec 13;13(24):6260. doi: 10.3390/cancers13246260 (PMC8699376; doi:10.3390/cancers13246260)
Supplement: Supplementary file 1 [file cancers-13-06260-s001.zip › cancers-1472454-supplementary.pdf]

Supplementary Table S1. Overview of the 29 patients that reached 10-year OS.

Patient	Recurrence	Age	Resection margin	Lymph node status	Tumor differentiation	HA reconstruction	PV reconstruction
1	yes	49	R0	N0	moderate	No	No
2	yes	75	R0	N0	moderate	No	No
3	yes	77	R0	N1	poor	No	No
4	yes	71	R0	N0	moderate	No	No
5	no	64	R0	N1	moderate	No	No
6	no	72	R0	N0	well	No	No
7	no	55	R0	N1	poor	No	No
8	no	60	R0	N0	well	No	No
9	no	57	R0	N0	well	No	Yes
10	no	41	R0	N0	moderate	No	No
11	no	63	R0	N0	moderate	No	Yes
12	no	60	R1	N0	moderate	No	Yes
13	no	77	R0	N0	moderate	No	No
14	no	62	R0	N0	poor	No	No
15	no	69	R0	N0	well	No	No
16	no	65	R0	N0	moderate	NA	Yes
17	no	52	R1	N0	well	NA	Yes
18	no	62	R0	N0	well	NA	Yes
19	no	46	R0	N0	moderate	NA	No
20	no	38	R0	N0	moderate	NA	No
21	no	63	R0	NA		No	No
22	no	61	R0	N1	well	No	No
23	no	66	R0	N0	well	No	No
24	no	65	R0	N0	well	NA	No
25	no	49	R0	N0	well	NA	Yes
26	no	75	R0	N0	moderate	No	No
27	no	61	R0	N0	moderate	Na	No
28	no	66	R0	N0	moderate	Na	No
29	no	68	R0	N0	moderate	NA	No

NA—not available, PV—portal vein, HA—hepatic artery.
